# Supplementary material for: Microisolation of Spatially Characterized Single Populations of Neurons for RNA Sequencing from Mouse and Postmortem Human Brain Tissues
Source: J Clin Med. 2023 May 5;12(9):3304. doi: 10.3390/jcm12093304 (PMC10179294; doi:10.3390/jcm12093304)
Supplement: Supplementary file 1 [file jcm-12-03304-s001.zip › jcm-2288979-supplementary.pdf]

Rapid Antibody staining protocol

For antibody staining, a shortened protocol based on the Vector Labs ImmPress HRP reagent kit (anti-goat MP-7405) was used, with the Vector Labs ImmPACT NovaRed chromogen serving as the peroxidase substrate (SK-4805).

i. Perform stepdown equilibration steps as indicated in the Sample Preparation for Laser Capture Microdissection section (Methods).
ii. Rinse with PBS ~500 µl per slide.
iii. Blocking buffer (from ImmPress kit, NHS blocking buffer), aliquot ~500 µl per slide to 1.5ml tube, add RNase Inhibitor. Add dropwise to each slide and incubate 5 min at RT.
iv. Remove blocking buffer but do not rinse with PBS, to fully remove, you can dab with a small Kimwipe to the edge of slide.
v. Add Primary antibody made up in diluent buffer (1X PBS with 2% normal goat serum, 0.01% Triton X-100 and 1 µl Superase RNAse Inhibitor per ml). Test the concentration needed for shortened protocol for adequate staining, note higher concentrations will be necessary compared to longer protocols which will also increase background staining.
vi. Incubate slides with primary antibody solution for 25 min at RT in a humidified chamber to reduce evaporation.
vii. Rinse with PBS 2x ~500 µl per slide.
viii. Drop cold (4 ˚C) secondary antibody solution onto slide with 1 ml pipette (pre-made, aliquot into 1.5ml tube and add RNase Inhibitor) and incubate at RT for 20 min in humidified chamber to reduce evaporation.
ix. During secondary antibody staining, prepare the NovaRed solution per manufacturer's specifications and keep at 4 ˚C until ready to use.
x. Rinse with PBS 2x ~500 µl per slide.
xi. Add NovaRed solution and incubate 5 min at RT (or until staining is at the level desired ~2-15 min).
xii. Rinse with PBS 2x ~500 µl per slide.
xiii. Air dry slide(s) at RT for ~5 min or until slide is completely dry (can be done in a fume hood or with a fan on low speed to shorten air drying time).
xiv. Go directly to LCM or store under desiccant at -80 ˚C until ready for LCM.

Rapid Nissl staining protocol

i. Perform stepdown equilibration steps as indicated in the Sample Preparation for Laser Capture Microdissection section (Methods).

ii. Rinse slides with cold (4 ˚C) PBS with optional 1 µl RNase Inhibitor added per ml of solution.

iii. Add Nissl stain solution (49.44 mM sodium acetate, 3.6 mM glacial acetic acid, 0.1% thionin, Sigma) for ~ 30 sec-1min at RT.

iv. Rinse slides 2x with cold (4 ˚C) PBS with optional 1 µl RNase Inhibitor added per ml of solution.

v. Air dry slide(s) at RT for ~5 min or until slide is completely dry (can be done in hood or with fan on low speed to shorten air drying time).

vi. Go directly to LCM directly to LCM or store under desiccant at -80 ˚C until ready for LCM.
